# Disruption of Bone Zinc Metabolism during Postnatal Development of Rats after Early Life Exposure to Cadmium

**DOI:** 10.3390/ijms21041218

**Published:** 2020-02-12

**Authors:** Sana Boughammoura, Safa Ben Mimouna, Marouen Chemek, Agnes Ostertag, Martine Cohen-Solal, Imed Messaoudi

**Affiliations:** 1 LR11ES41: Laboratoire de Recherche Génétique, Biodiversité et Valorisation des Bioressources, Institut de Biotechnologie, Université de Monastir, Monastir 5000, Tunisia; benmimounasafa@yahoo.fr (S.B.M.); chemekm@yahoo.com (M.C.); imed_messaoudi@yahoo.fr (I.M.); 2 Bioscar Inserm U1132 and université de Paris, hôpital Lariboisière, 75010 Paris, France; agnes.ostertag@inserm.fr (A.O.); martine.cohen-solal@inserm.fr (M.C.-S.)

**Keywords:** bone development, Cadmium, Zinc, Zinc transporters, gestation and lactation

## Abstract

This current study was conducted to investigate whether bone tissue impairment induced by early life exposure to cadmium (Cd) during postnatal development could result from disruption to zinc (Zn) metabolism. For this reason, the offspring from mothers receiving either tap water, Cd, Zn or Cd + Zn during gestation and lactation periods were euthanized at PND21 and PND70. At the end of the lactation period (PND21), our results showed that exposure to Cd increased Cd accumulation and Zn depletion in the femur. Furthermore, calcium (Ca) level was reduced. At the molecular level, Cd induced an increase of MT-1 expression and caused an upregulation of ZIP2 accompanied with a down-regulation of ZnT5. Runx2, ALP, colα-1 and Oc mRNA levels were also decreased. In plasma, IGF-1 and osteocalcin concentrations were decreased. Further, Cd altered femoral growth by generating changes in the growth plate. Consequently, the toxic effect of Cd persisted at adult age (PND70) by decreasing bone volume (%BV/TV), bone mineral density (BMD) and Ca content and by increasing trabecular separation (Tb.Sp) in the distal femur. Interestingly, Zn supply provided total or partial corrections of several toxic effects of Cd. These data suggest that the increases of Zn bioavailability as well as the reduction of Cd accumulation in the femur following the changes in ZIP2 and ZnT5 expression are part of the mechanism involved in Zn protection against Cd toxicity on bone tissue.

## 1. Introduction

Cadmium (Cd) is a toxic substance that is widely distributed in the environment and has a long biological half-life in organs. Kidneys, liver, respiratory, cardiovascular systems, and bone are the most important target organs for Cd toxicity [1]. Several epidemiological studies have shown that relatively low exposure to this metal can lead to severe skeletal damage [2,3,4]. In addition, it has been reported that prolonged exposure to Cd may cause disruption in bone markers regulation [5] and in calcium metabolism [6], inducing osteomalacia [7] and a high risk of osteoporosis [8]. Studies have shown that Cd administration during gestation and lactation periods [9] leads to its accumulation in the mammary glands of female rodents. The same findings indicate that Cd transfer through maternal milk represents the primary route of offspring exposure. Consequently, a number of neuro-toxicological and behavioral effects were observed during postnatal development [10].

Some researchers have shown that the Cd toxic effect can be mediated by a disruption in zinc (Zn) metabolism [11,12]. Zn plays an important role in the growth, the development and the maintenance of bones [13,14]. Further, it acts on the activity and structure of various enzymes as well as osteogenic factors involved in the endochondral bone formation of long bones [15]. Moreover, Zn supports collagen formation and phosphorus fixation in bone tissue. Earlier study suggest that Zn deficiency may have irreversible effects in pups during bone postnatal development by inducing skeletal growth retardation [16] and by decreasing osteogenic processes [17], making the bone more sensitive to Cd [18]. However, a Zn supplement diet is able to provide protection against Cd toxicity and can also reduce its absorption and accumulation [19,20].

According to previous data, the interactions between Cd and Zn can take place in all stages of Zn metabolism (intestinal absorption, distribution, accumulation and excretion) [21,22]. In the other hand, it has been established that Zn transporters and Zn storage protein, such as metallothioneins (MTs), Zrt- and Irt-like proteins (ZIP), and Zn transporters (ZnT), are required for the activation of many enzymes involved in bone mineralization [23]. Several studies indicate that Cd gains access to cells by mimicking Zn at the site of Zn transporters [24,25]. Our previous study [26] implies that the downregulation of ZnT as well as the overexpression of ZIP transporters in the mammary gland of lactating rat and in the intestines of their offspring play a major role in Cd accumulation and Zn redistribution in pup’s tissues. In a further study, we have demonstrated that the toxic effects of Cd observed during development are mediated by the disruption of maternal Zn metabolism during gestation period, causing Zn depletion in fetuses and continues to become more pronounced during lactation [26]. Similar findings were reported in our recent study which showed that Cd exposure during gestation period affects maternal Zn metabolism, leading to Zn depletion and perturbation of prenatal bone formation [27]. Therefore, the current study is a continuous report, in which we used the same conditions of our previous work [27] in order to investigate the involvement of the disruption of Zn metabolism in Cd-induced bone tissue impairment in offspring and its consequences at adult age. Also, we provided insight to the possible mechanism underlying the protective effect of Zn against Cd toxic effect on bone tissue.

## 2. Results

### 2.1. Effects on Postnatal Bone Formation

#### 2.1.1. Cadmium and Zinc Concentrations

Compared to the control group (Table 1), exposure to Cd during pregnancy and lactation resulted in a significant increase in Cd accumulation (*p* < 0.01) and reduced Zn level (*p* < 0.01) in the femur of pups at PND21. However, combined treatment by Cd and Zn seemed to partially reverse the effects observed in Cd-exposed animals. Indeed, this treatment caused a significant decrease (*p* < 0.01) in Cd concentrations and restored Zn content (*p* < 0.01) in the femur compared to the Cd group.

#### 2.1.2. Body Weight Progression (BWP), Relative Femur Weight and Craniocaudal Length (CCL)

**Figure 1:**
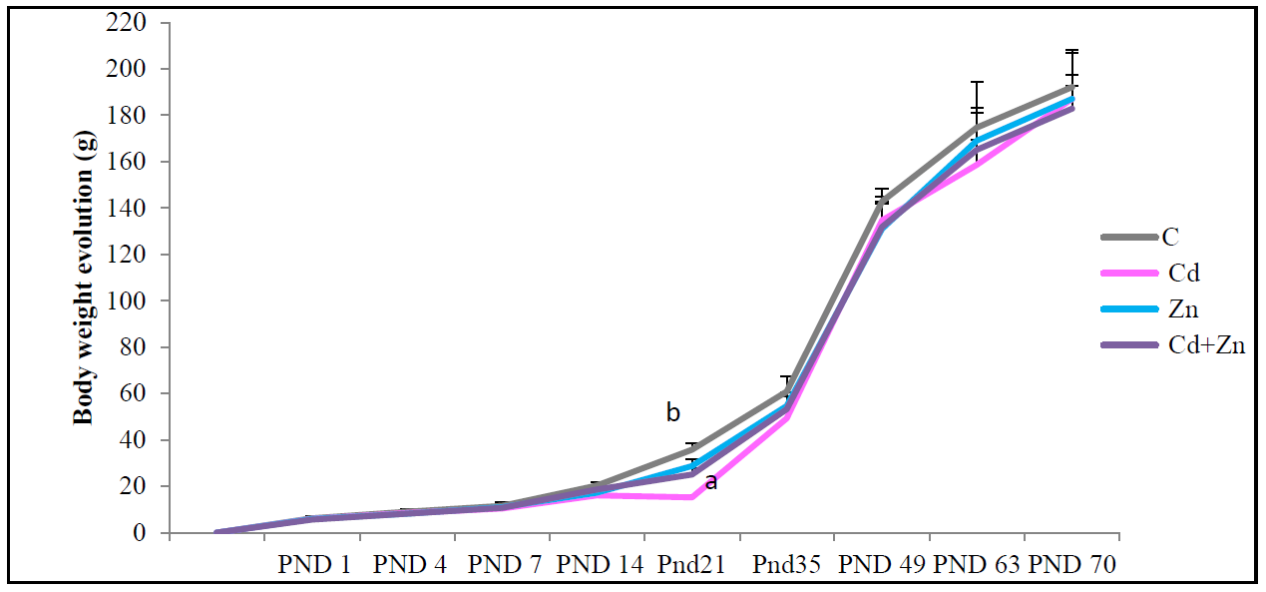
Body weight evolution of rat pups from control females and pups from females exposed to cadmium (50 mg/L Cd as CdCl2) and/or zinc (60 mg/L Zn as ZnCl2) during gestation and lactation. Values are expressed as mean ± SEM from 12 to 18 animals from each group (2–3 male per litter was measured). a (*p* < 0.05): significant difference compared to the control group (C); b (*p* < 0.05): significant difference compared to the Cd group (Cd).

As shown in Figure 1, exposure to Cd, during pregnancy and lactation, significantly decreased (*p* < 0.05) the pups’ body weight at PND21 compared with the control group and those in the Zn group. The co-treatment of Zn with Cd entirely reversed the Cd-induced body weight decrease (*p* < 0.05) observed at PND 21. After lactation no significant difference in BWP was observed until PND70.

Results in Table 2 show that Cd and/or Zn exposure during gestation and lactation did not affect the relative femur weight at PND21 and PND70. However, Cd exposure significantly decreased (*p* < 0.01) craniocaudal length (CCL) of the male offspring at PND 21 when compared to the control and Zn animals. Indeed, the CCL of animals exposed simultaneously to Cd and Zn was almost similar to control values at PND 21 and was significantly higher (*p* < 0.05) compared to the Cd group.

#### 2.1.3. Femur Calcium Analysis

**Figure 2:**
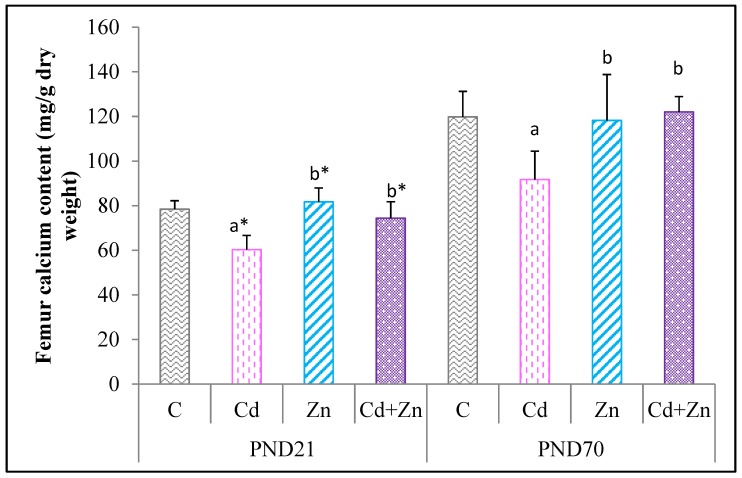
Femur calcium content of rat pups from control females and pups from females exposed to cadmium (50 mg/L Cd as CdCl2) and / or zinc (60 mg/L Zn as ZnCl2) during gestation and lactation. Means ± SEM from six samples in each groups; a* (*p* < 0.01); a (*p* < 0.05): significant difference compared to the control group (C); b* (*p* < 0.01); b (*p* < 0.05): significant difference compared to the Cd group (Cd).

Figure 2 shows that Cd exposure during pregnancy and lactation caused a significant decrease in Ca content in the femoral tissue of pups at PND21 (*p* < 0.01) and at PND70 (*p* < 0.05) compared with the control group and the Zn group. The co-treatment of Zn with Cd (Cd + Zn group) restored the Cd-induced Ca content decrease (*p* < 0.05) observed in Cd-treated group at PND 21 and PND70.

#### 2.1.4. Ash Weight to Dry Weight Ratio (AW/DW), Nonorganic (%CNO), and Organic Content (%CO)

Table 3 shows the results for %CO, %CNO, AW/DW. Indeed, no significant difference was noted between the different groups at PND21 and PND70.

### 2.2. Effect on IGF-1 and Osteocalcin Levels

**Figure 3:**
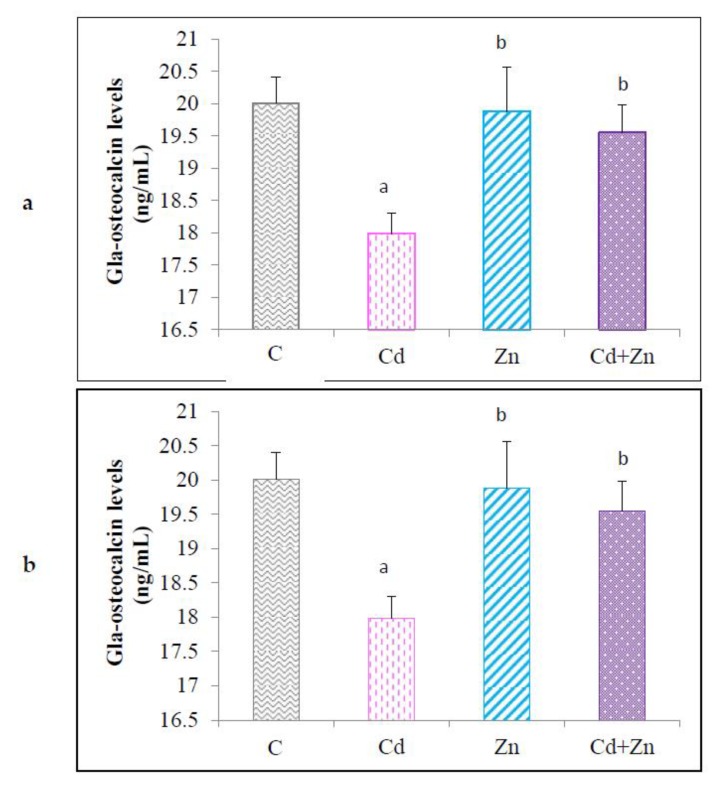
IGF-1 (**a**) and Osteocalcin (**b**) concentrations in the plasma of rat pups (PND21) from control females and pups from exposed to cadmium (50 mg/L Cd as CdCl2) and / or zinc (60 mg/L Zn as ZnCl2) during gestation and lactation. Means ± SEM from six samples in each groups; a* (*p* < 0.01); a (*p* < 0.05): significant difference compared to the control group (C); b* (*p* < 0.01); b (*p* < 0.05): significant difference compared to the Cd group (Cd); c (*p* < 0.01): significant difference compared to the Zn group (Zn).

Maternal exposure to Cd during gestation and lactation periods led to a significant (*p* < 0.05) decrease of IGF-1 levels in the plasma of male pups at PND21 compared with the animals in the control group and those treated only by Zn (Figure 3a). Simultaneous treatment with Cd and Zn showed a reduction in the effect of the toxic metal. Indeed, IGF-1 levels in the Cd + Zn group were significantly higher (*p* < 0.05) compared to Cd group, but remained lower than that observed in the control group and the Zn group (*p* < 0.05).

The results relating to the osteocalcin plasma level of pups at PND21 are presented in Figure 3b. By exposing the mother rats to Cd during pregnancy and lactation, the plasma concentration of osteocalcin showed a significant decrease (*p* < 0.01) in pups at PND 21 compared to controls animals and those treated with Zn alone. However, simultaneous treatment with Cd and Zn significantly increased (*p* < 0.05) the osteocalcin levels in the plasma of offspring compared to the Cd group.

### 2.3. Femur Histomorphometry at PND21

#### 2.3.1. Total Femur Length, Femur Width and Femur Area

**Figure 4:**
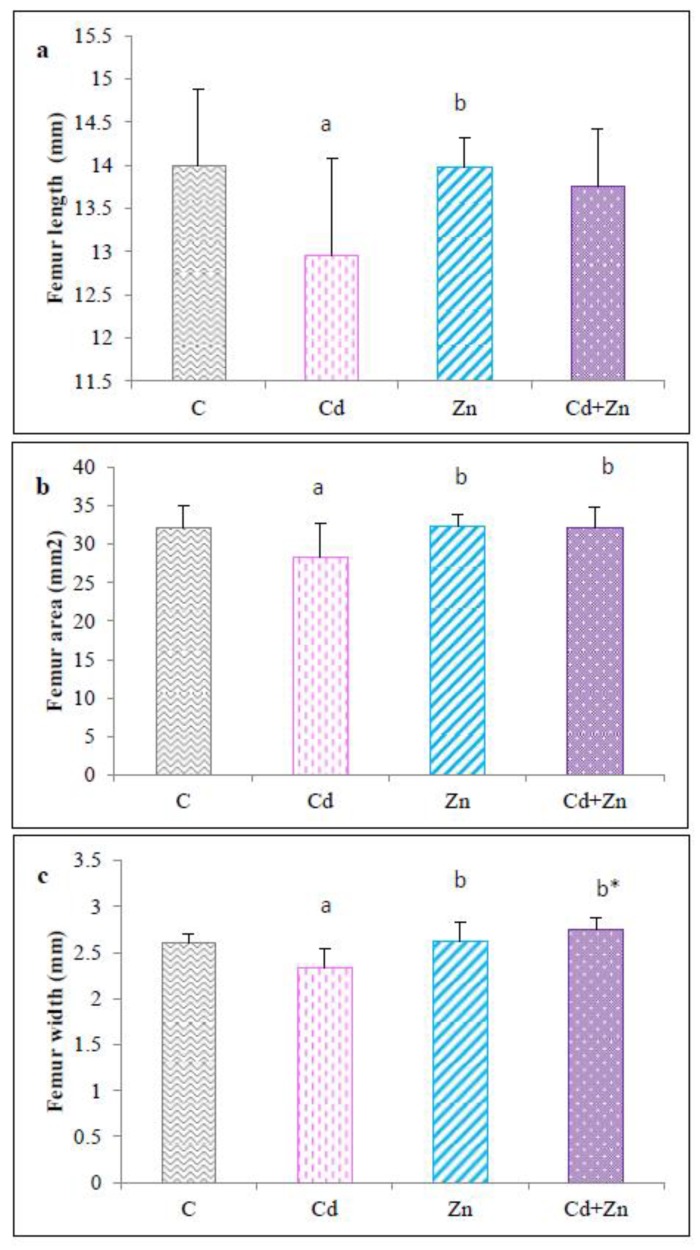
Femur length (**a**), femur area (**b**), and femur width (**c**) of rat pups (PND21) from control females and pups from females exposed to cadmium (50 mg/L Cd as CdCl2) and / or zinc (60 mg/L Zn as ZnCl2) during gestation and lactation. Means ± SEM from six samples in each groups; a (*p* < 0.05): significant difference compared to the control group (C); b* (*p* < 0.01); b (*p* < 0.05): significant difference compared to the Cd group (Cd).

Exposure to Cd during gestation and lactation resulted in a significant decrease (*p* < 0.05) in femoral length (Figure 4a), width (Figure 4b), and area (Figure 4c) in pups at PND21 from Cd-treated mothers compared to the control group and Zn group. However, simultaneous treatment with Cd and Zn corrected the decrease in total area (*p* < 0.05) and width (*p* < 0.01) compared with the Cd group.

#### 2.3.2. Diaphysis Length and Diaphysis Area

**Figure 5:**
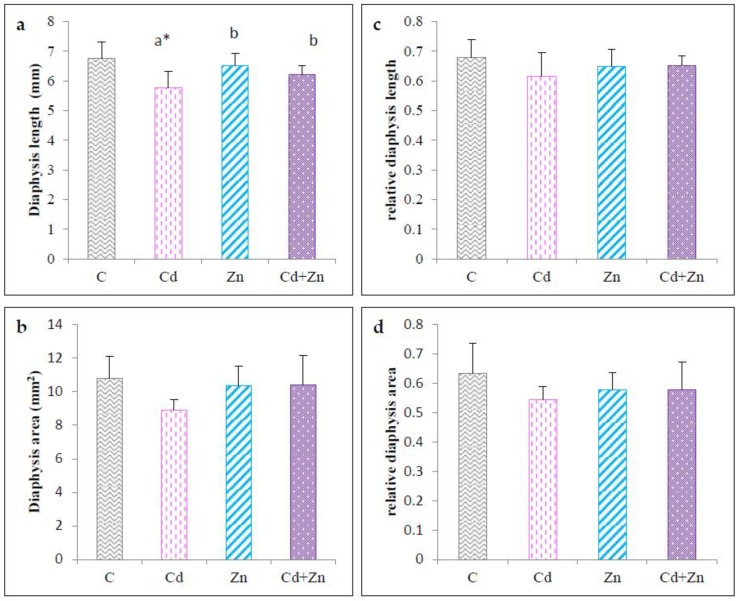
Length diaphysis (**a**), diaphysis area (**b**) relative length diaphysis (**c**) and relative diaphysis area (**d**) of femur of rat pups (PND21) from control females and pups from females exposed to cadmium (50 mg/L Cd as CdCl2) and / or zinc (60 mg/L Zn as ZnCl2) during gestation and lactation. Means ± SEM from six samples in each groups; a* (*p* < 0.01); a (*p* < 0.05): significant difference compared to the control group (C); b (*p* < 0.05): significant difference compared to the Cd group (Cd).

For the central part (the diaphysis), the results are shown in Figure 5. A significant decrease (*p* < 0.05) in the length of the diaphysis (Figure 5a) and the area (Figure 5b) of femurs at PND21 was noted in the Cd-exposed group compared to the control and Zn group. A total correction (*p* < 0.05) of the diaphysis length was recorded in the femur of pups from animals treated simultaneously with Cd+Zn compared with the Cd group. In addition, no significant difference was noted regarding the relative diaphysis length (Figure 5c) and the relative diaphysis area (Figure 5d) between the four groups.

#### 2.3.3. Length, Area, Relative Length and Relative Area of the Hypertrophic and Proliferative Zones

**Figure 6:**
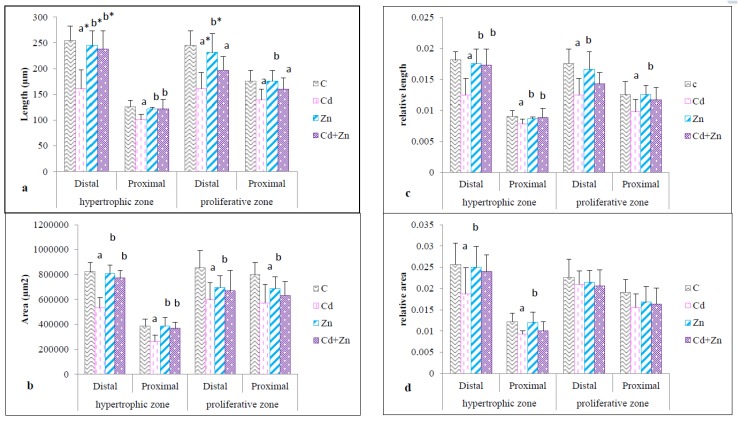
Length (**a**), area (**b**), relative length (**c**) and relative area (**d**) of the hypertrophic and proliferative zones of the femur of rat pups (PND21) from control females and pups from females exposed to cadmium (50 mg/L Cd as CdCl2) and / or zinc (60 mg/L Zn as ZnCl2) during gestation and lactation. Means ± SEM from six samples in each groups; a* (p < 0.01); a (*p* < 0.05): significant difference compared to the control group (C); b* (*p* < 0.01); b (*p* < 0.05): significant difference compared to the Cd group (Cd).

As shown in Figure 6, whether it was the distal or proximal epiphyses in the femur of pups, Cd exposure during pregnancy and lactation resulted in a significant decrease (*p* < 0.05) in the length of the hypertrophic zone and the proliferative zone (Figure 6a) as well as their areas (*p* < 0.05) (Figure 6b) compared with the control and Zn group. In the Cd + Zn group, a restoration (*p* < 0.05) of the length and area of the hypertrophic zone was observed compared to the Cd group. However, this type of treatment did not show a significant difference in the proliferative zone. As shown in Figure 6c, the relative length of the hypertrophic and the proliferative zones (proximal and distal) was significantly reduced (*p* < 0.05) following exposure to Cd compared with the control and the Zn group. Co-treatment by Cd and Zn showed a total correction only of the relative length of the hypertrophic zone (*p* < 0.05). Compared to the control, the relative area of the hypertrophic zone (proximal and distal) (Figure 6d) showed a significant decrease (*p* < 0.05) by exposing animals to Cd. In the presence of Zn (the Cd + Zn group) no correction was observed compared to the Cd group. In addition, at the level of the proliferative zone no difference was noted, either at the distal or proximal epiphyses, between the four studied groups.

#### 2.3.4. Trabecular Bone Volume (BV/TV)

**Figure 7:**
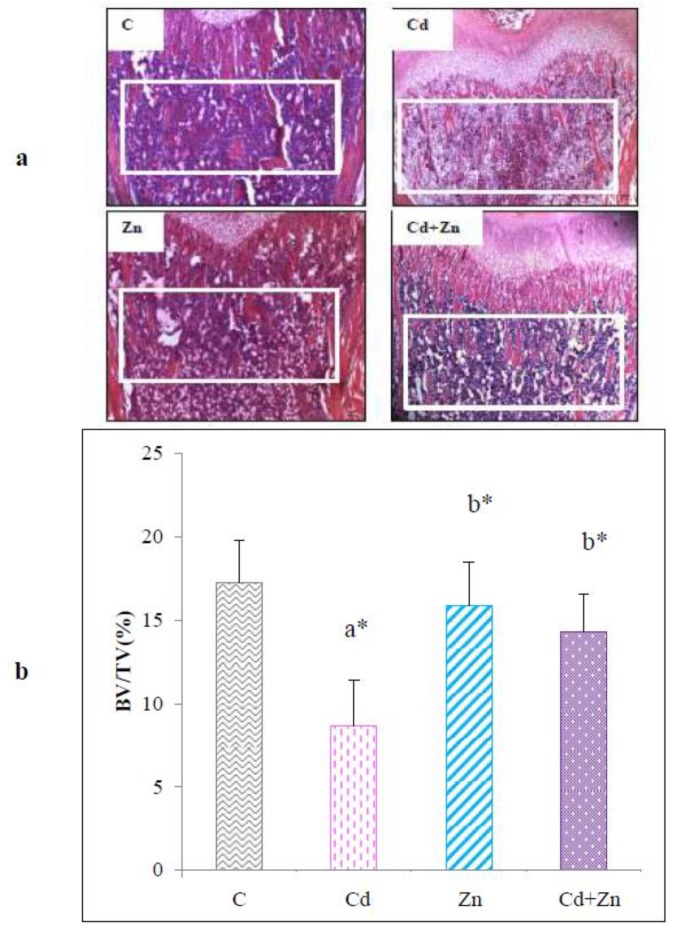
Histological section (**a**) of distal femur of rat pups (PND21) from control females and pups from females exposed to cadmium (50 mg/L Cd as CdCl2) and/or zinc (60 mg/L Zn as ZnCl2) during gestation and lactation (scale bar: 400 μm). Bone mineral volume (%BV/TV) was evaluated by counting trabecular bone on the region of interest (**b**). Means ± SEM from six samples in each groups; a* (*p* < 0.0001): significant difference compared to the control group (C); b* (*p* < 0.01): significant difference compared to the Cd group (Cd).

Bone mineral volume was determined at the level of the trabecular bone by histological analysis of femur of pups at PND 21 (Figure 7a). The results are reported in Figure 7b. Indeed, a highly significant decrease (*p* < 0.0001) in BV/TV was noted at the distal femur following maternal exposure to Cd during pregnancy and lactation compared to the control group and Zn group (*p* < 0.01). However, the combined treatment with Cd + Zn significantly reversed (*p* < 0.01) the reduction of BV/TV observed in Cd group.

### 2.4. Gene Expression

#### 2.4.1. Effect on Zn Transporters mRNA

**Figure 8:**
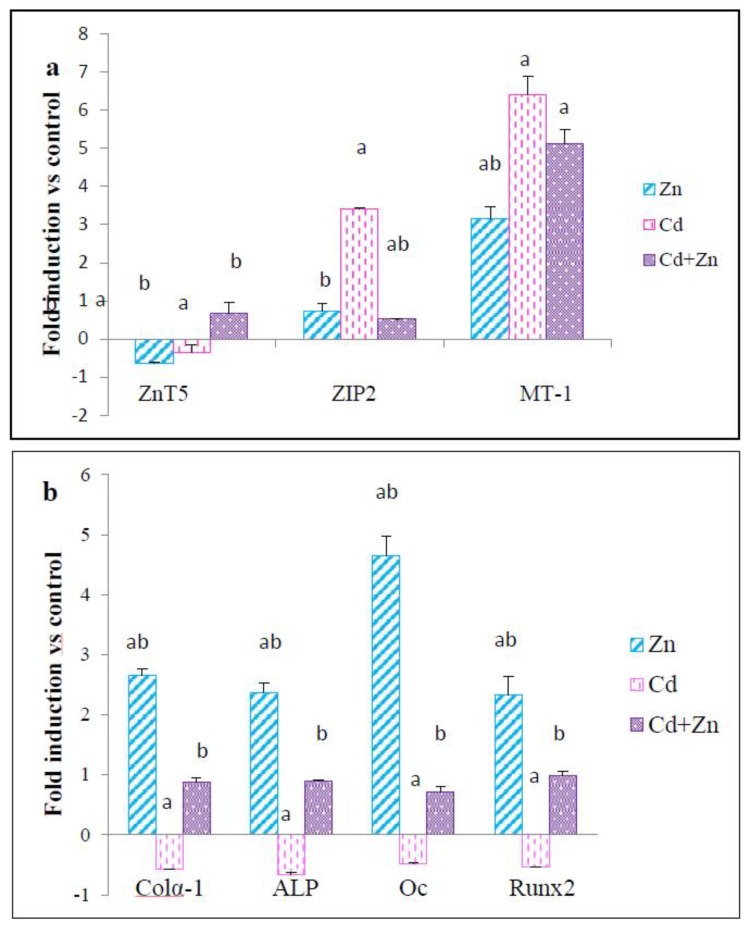
Quantitative real-time PCR expression of genes encoding Zn transporters (MT-1, ZnT5 and ZIP2) (**a**) and of specific bone differentiation markers (**b**) in femur tissues of rat pups (PND21) from control females and pups from females exposed to cadmium (50 mg/L Cd as CdCl_2_) and / or zinc (60 mg/L Zn as ZnCl_2_) during gestation and lactation. Data represent expression levels with respect to control samples. a (*p* < 0.05): significant difference compared to the control group (C); b (*p* < 0.05): significant difference compared to the Cd group (Cd).

The effects of Zn and/or Cd exposure on expression of genes encoding Zn transporters (ZnT5, ZIP 2, and MT-1) in femur of pups at PND21 are reported in Figure 8a. Upregulation of MT-1 gene expression (*p* < 0.05) in the femur of offspring was noticed under Cd and/or Zn effects compared to controls. The expression pattern of mRNA encoding the ZIP2 and ZnT5 transporter, following Cd exposure, was marked by a significant increase (*p* < 0.05) in the expression level of ZIP2 and a significant decrease (*p* < 0.05) of ZnT5 gene expression compared to the control group. Treatment with Cd + Zn reversed the toxic effects of this metal. Indeed, this correction was marked by an upregulation of the ZnT5 gene (*p* < 0.05) while maintaining a high expression level of the ZIP2 gene (*p* < 0.05) compared to the Cd group.

#### 2.4.2. Effect on mRNA of Specific Bone Differentiation Markers

The results presented in Figure 8b show that Zn treatment increased significantly (*p* < 0.05) the mRNA levels of colα1, ALP, osteocalcin, and Runx 2 in the femur at PND21 compared to pups from control mothers (*p* < 0.05). Cd exposure significantly depressed the expression of the four studied genes with respect to control animals. Interestingly, Cd and Zn treatment restored Cd-induced gene downregulation. In fact, the expression level of colα1, ALP, osteocalcin, and Runx 2 genes were found to be upregulated (*p* < 0.05) compared to the Cd group.

#### 2.4.3. Bone Mass and Architecture

**Figure 9:**
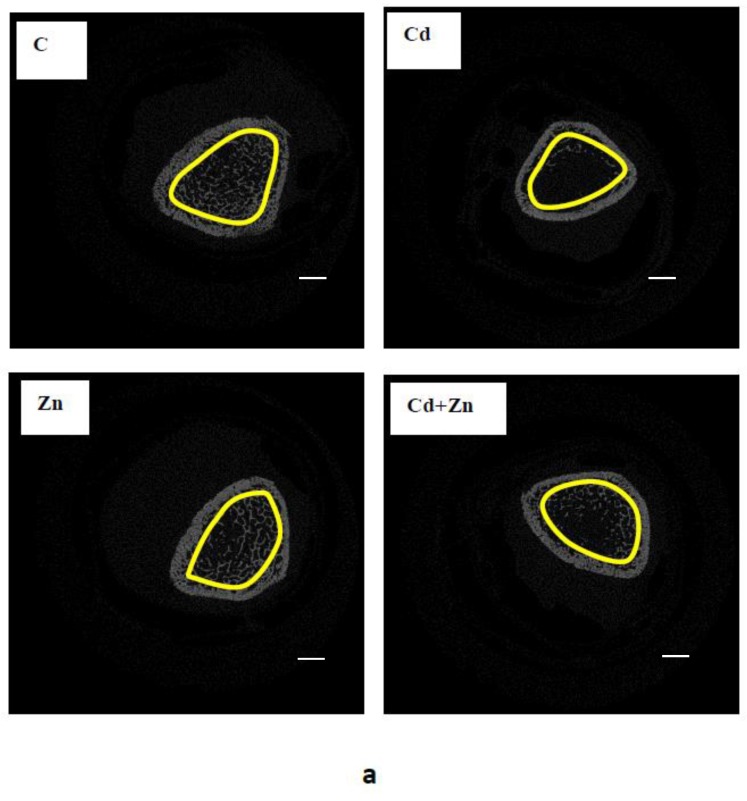

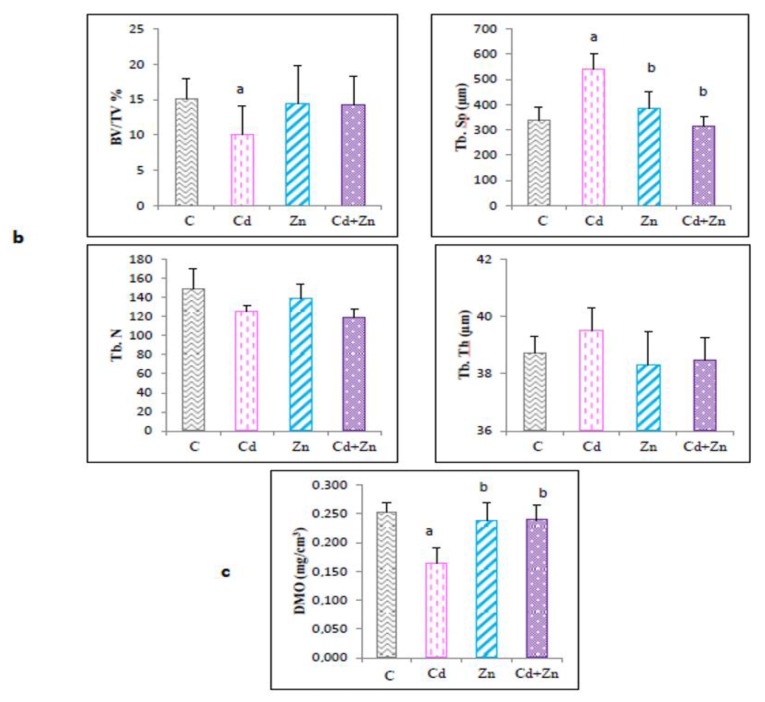
(**a**) Cross-sectional micro-computed tomography reconstruction was performed on distal femur ((scale bar: 1mm) of rats (PND70) from control females and rats from females exposed to cadmium (50 mg/L Cd as CdCl2) and / or zinc (60 mg/L Zn as ZnCl2) during gestation and lactation, (**b**,**c**) Trabecular bone volume (%BV/TV), trabecular number (Tb.N), trabecular thickness (Tb.Th), trabecular separation (Tb.Sp) were measured and BMD was determined on femur sections. Values are expressed as mean ± SEM from 7 samples. a (*p* < 0.05); significant difference compared to the control group (C); b (*p* < 0.05); significant difference compared to the Cd group (Cd).

The results relating to bone microarchitecture at the distal femur of the rats at PND 70 have been described in Figure 9a,b. Mothers exposure to Cd during pregnancy and lactation caused a significant decrease (*p* < 0.05) in bone volume (%BV/TV) and in trabecular separation (Tb, Sp) in adult rats at PND70 compared with the control (Figure 9b). Simultaneous treatment with Cd + Zn showed a total restoration (*p* < 0.05) of Tb, Sp, however, no significant difference was noted regarding the %BV/TV compared to the Cd group. The results concerning the trabecular number (Tb. N) and trabecular thickness (Tb. Th) (Figure 9b) did not show a significant difference between the different groups. The bone mineral density (BMD) measurement at the distal femur was shown in Figure 9c. These results show a significant (*p* < 0.05) decrease in BMD in the Cd-exposed animals compared to the control and the Zn group. However, combined treatment (Cd + Zn) completely restored the BMD compared to the group exposed to Cd.

## 3. Discussion

In a recent study [27], we described for the first time Zn depletion and disruption of prenatal bone formation induced by Cd exposure during gestation. The current study is a continuous report in which we studied the effects on bone development in pups of rats from mothers treated by Cd and/or Zn during gestation and lactation and its consequences at adult age (PND70) in order to elucidate the mechanism likely to be involved in these effects.

It has been suggested that relatively low exposure to Cd causes significant damage to bone tissue [2,4]. Our results showed a Cd accumulation in the femur of pups at PND21 after maternal exposure to Cd during pregnancy and lactation. The detectable Cd in the femur of offspring was also in agreement with previous investigations, which confirms the importance of lactational Cd transfer [26]. At the same time, Cd fixation has been associated with a significant decrease in Zn contents in the femur of pups. The same results have been reported by Chemek et al. [26] which show that Cd accumulation affects Zn content in the testes of offspring from mothers treated with Cd during pregnancy and lactation. Our previous study [22] implies that the downregulation of ZnT and the overexpression of ZIP transporters in the mammary gland of lactating rats and in the intestine of their offspring play a major role in Cd accumulation and Zn redistribution in tissues of their offspring. On the basis of these results, the effect of Cd exposure on Zn depletion in the femurs of pups, could be attributed not only to the accumulation of metal in the mammary gland during lactation, but also to the action of Cd on Zn transporters leading to minimized Zn transfer from maternal circulation to milk. On the other hand, we noted that Cd accumulation in femurs induced a significant decrease in the Ca content in pup rats (PND21). Ohrvik et al. [28] attributed the Cd-induced Ca disturbances in lactating mammary glands of mice to the ability of this metal to interfere with Ca channels or bind to Ca transporters in the mammary gland. From this data, we can suggest that the Ca decrease induced by Cd exposure in the femur of offspring may be due to a disruption of maternal Ca metabolism causing a decrease in Ca transfer via the mammary gland to pups tissues.

Another study indicates that Zn homeostasis in the body is ensured by the regulation of its absorption and excretion rate involving storage proteins such as MTs [29] and two members of the transport protein family (ZIPs and ZnTs). In order to elucidate the underlying mechanism by which Cd induces its toxicity, we examined the expression of genes encoding Zn transporters in the femur. In addition, several authors have noted that ZIP2 and ZnT5 protein regulates Zn levels in the femur [15,30]. Referring to data from our recent study [27], we have previously showed that maternal Cd exposure during gestation impairs Zn hemeostasis in the bone tissue of fetuses. The same patterns of gene expression have been observed in the femur of pups at PND21 from Cd-treated mothers during pregnancy and lactation. Indeed, we have shown a significant increase in gene expression encoding MT-1. In addition, exposure to Cd strongly induced overexpression of ZIP2 and downregulation of ZnT5. A similar result was also noted in mammalian testes and kidneys [31]. We may speculate that Zn depletion, caused by Cd in the femur, is countered by the increased expression of ZIP2, a Zn importer, and by the downregulation of ZnT5, a Zn exporter, thus leading to Cd intracellular accumulation.

It has been well established that the level of gene expression encoding differentiation markers must be regulated to allow mature bone formation by the osteoblast [32,33]. The present study demonstrated that Cd exposure lead to a downregulation of Runx2 as well as of ALP, colα-1 and Oc. The cellular changes generated are associated with Zn depletion in the femur of offspring at PND21 as previously reported in several findings [5,17,27].

All the changes noted in our study are therefore in favor of Cd toxic effects leading to a femoral growth disturbance in offspring. The results of exposure to Cd during pregnancy and lactation showed an inhibition of the different morphometric parameters measured (total length, total surface, width, length and surface of the hypertrophic and proliferative zone, length and surface of diaphysis) in offspring at PND21. These changes were accompanied by a decrease in bone volume (%BV/TV) compared to animals from the control group. These results are in accordance with previous findings which demonstrated that Cd alone or combined with other metals such as lead and arsenic decrease the length of the hypertrophic and proliferative zone, thus leading to a decrease in the length of the growth plate [34]. Moreover, bibliographic data indicates that Zn deficiency disrupts growth and skeletal maturation and leads to a decrease in the availability of precursors for differentiation into hypertrophic chondrocytes and consequently the alteration of bone mass [16,35].

Furthermore, Zn deficiency is associated with metabolic disorders of a wide range of hormones, cytokines, and enzymes involved in bone growth and development [36]. Accordingly, the current study revealed that Cd exposure significantly decreases the level of IGF-1 in plasma in offspring at PND21 compared with the animals in the control group. This protein (IGF-1) is strongly involved in bone growth. Sun et al. [37] noted a decrease in gene expression of IGF-1 in the femur of young rats following a Zn-deficient diet and they attributed Zn deficiency-induced alterations in bone metabolism to the reduction of IGF-1 expression. Thus, the decrease in body weight and craniocaudal length of pups observed at PND 21 may be correlated with the decrease in plasma concentration of IGF-1 which is in agreement with other studies [38,39]. In addition, we noted a significant decrease in osteocalcin concentration in the plasma of the Cd-treated group at PND21 compared to the control group. There are several studies that report the role of Oc as an osteogenic marker in the final stage of osteoblastic differentiation [40]. Correspondingly, it has been reported in rats that Oc decrease in serum is associated to Zn-diet deficiency [12,41].

To our knowledge, no recent study has evaluated the effect of maternal exposure to Cd during pregnancy and lactation on bone tissue in rats at adult age. It has been reported that bone microstructure (or μCT) is a critical parameter for assessing bone strength [42]. Our study showed a significant decrease in BMD as well as in %BV/TV parameters accompanied by a significant increase in the trabecular separation (Tb.Sp) in the distal femur of adult rats at PND70. Likewise, several authors have noted similar results by exposing 60-day-old rats to 50mg Cd/L for three months [43] or following chronic exposure to male rats during development [44,45]. We think that the decrease in BMD noted in our study partly explains the decrease in Ca content in the femurs of adult rats at PND70. In the present study, we suggest that growth retardation, and the histomorphometric and microstructural changes noted in the femur of rats during postnatal development, induced by maternal exposure to Cd, are strongly due to the depletion of Zn in these animals. Our suggestion is in accordance with the new observation on the mechanism of Cd-induced bone toxicity described in a recent study [27]. Here, we suggest that Cd can alter bone development in offspring by interfering with Zn homeostasis, which in turn leads to an alteration of microstructure in adult age due to the accumulation of Cd in the femur during lactation.

Zn is one of the most important nutritional factors influencing the metabolism and toxicity of Cd [21]. In bone tissue, Zn plays an essential role in bone growth, development and maintenance [46]. For years, we have been interested to study the protective effect of Zn against Cd-induced toxicity in various organs [22,26,28,47]. The present work showed that the combined treatment by Cd and Zn during gestation and lactation reduced Cd accumulation and increased Zn content in femur of pups at PND21. The protective effect of Zn may be due to the interaction between the two metals at the different stages of Zn metabolism and in femur itself. Brzoska and Moniuszko-Jakoniuk [21] have noted that the the co-treatment with Cd and Zn in the small intestine increases Zn distribution and accumulation in various organs, particularly the femur. Moreover, it has been shown that the decrease in Cd content could be due to its interaction with Zn transporters in the mammary gland, leading to less transfer of Cd from maternal circulation to milk which in turn leads to a decrease in Cd accumulation in the organism of their offspring [22]. Regarding Ca content, we noted a total restoration of Ca levels in the femur of pups at PND21. Brzoska et al. [48] reported that Zn supplementation in the presence of Cd prevents the Cd-induced decrease of Ca content in the tibia of rats.

At the cellular level, with the combined treatment, MT-1 gene expression was upregulated to protect against Cd toxicity in the femur of pups at PND21. The ability of the essential element to induce MTs which sequesters Cd had been suggested as the most likely mechanism [21]. With respect to Zn transporters, in the current study, ZnT5 was found to be upregulated while a persistently increased of ZIP2 levels were noticed in Cd + Zn-treated group compared to Cd-treated group. Our result suggests that, in bone tissue, the ameliorative effect of Zn against Cd accumulation is mediated, partly by the increase of Zn bioavailability and subsequently the induction of ZnT2 gene expression. Taken together, high levels of Zn with the upregulation of ZnT5 gene expression will promote the release of Cd outside the cell compartment thereby reducing these concentrations in the femur of pups at PND21. Previous study showed similar findings following simultaneous treatment with Cd and Zn in various organs [22,47]. On the other hand, an upregulation of the genes encoding for the matrix proteins (ALP, colα-1 and Oc) as well as for the Runx2 has been noted in femur of pups at PND21 with combined treatment. There is also evidence that insufficient Zn intake leads to the inactivation of ALP [49], and decreases the transcription of osteoblast differentiation genes via a downregulation of the Runx2 in MC3T3-E1 cells [50]. This emphasizes the fundamental role that Zn plays in gene transcription and it can be explained, in part, by the fact that Zn supply increases its bioavailability and thus enhances intracellular Zn levels, leading to the induction of differentiation marker gene expression.

Regarding the effects on bone histomorphometric, our results showed that simultaneous treatment with Cd and Zn during gestation and lactation partially correct the damage caused by Cd in growth plate. In fact, Zn supply leads to improve the proliferative and hypertrophic zone length in femur of pups at the end of the lactation (PND21). Further, we observed that Zn supply increased partially circulating IGF-1and Oc in the plasma of pups at PND21 compared to the Cd group. These results corroborate with those of several studies showing that Zn supply stimulate osteoblastic activity and reduce Cd-induced bone resorption [12,37]. The beneficial effect of Zn is reflected in the adult rat (PND 70) firstly by the recovery of Ca amount and also by a total restoration of BMD and a decrease in the Tb.Sp in the femur. Some authors have reported that a diet rich in Zn (60 mg/kg) is necessary to increase Ca content also to improve resistance and connectivity of the femoral tissue in young rats during development [3], while Zn deficiency (2 mg/kg) is associated with a decrease in bone strength [14], thus leading to a reduction in the volume of trabecular bone [16]. The beneficial effect of Zn supply on bone tissue observed in our study gives evidence that Zn supports bone formation to ensure the optimal development of bone tissue during postnatal development.

Like Zn, copper (Cu) is an essential trace element involved in various biological functions, and its homeostasis is controlled by proteins such as metallothionein (MTs) and membrane transporters. Zn contents can influence Cu absorption by competing for binding with MTs [51]. Further, it has been reported that Zn-rich diets decrease Cu absorption; this treatment is also used to treat Wilson’s disease [52]. According to the present study and due to the chemical and physical similarity between Cd, Zn and Cu, we may predict competitive interactions at binding sites on metal-binding proteins such as MTs, and Cu-Zn superoxide dismutase (SOD), or on metal influx/efflux transporters. Further studies will be necessary to better understand the implication of copper in the interaction between these metals.

In conclusion, our results imply that Cd exposure during gestation and lactation has an adverse effect on bone development. The toxic effect of Cd is, partly, caused by the disruption of Zn metabolism in offspring when Cd is being transferred through the milk, leading to the decreased transcription of gene markers involved in bone formation. This results in a disruption of growth as well as an alteration in femur development at the end of lactation. Consequently, the toxic effect of Cd persists at adult age and impairs femoral microstructure. The ameliorative effect of Zn in Zn metabolism can be considered as a probable molecular mechanism that explains the protective role of Zn against Cd-induced femur toxicity and bone structure alteration in the adult rat. Future additional studies are strongly required to better explore this molecular mechanism.

## 4. Materials and Methods

### 4.1. Animals and Experimental Design

A parental generation of male and female Wistar rats, purchased from a local supplier (CYPHAT, Tunisia), was subjected to a 2-week acclimatization period. The animals were housed in individual stainless steel cages at 23 ± 1 °C and exposed to a 12 h light-dark cycle. They had access to a standard rodent laboratory diet (SICO, Sfax, Tunisia) and drinking water ad libitum. The animals were housed according to the EEC 609/86 Directives regulating the welfare of experimental animals. After acclimatization, male and female rats were mated to obtain first-generation offspring. During mating, rats were separated after positive identification of a vaginal sperm plug, after which a designation of gestational day zero (GD0) was made. At GD0, pregnant rats were housed individually in plastic cages and randomly divided into four groups (*n* = 6). A control group of animals received tap water and three experimental groups received either Cd (50 mg/L Cd as CdCl2), Zn (60 mg/L Zn as ZnCl2) or Cd + Zn (50 mg/L Cd + 60 mg/L Zn) in their drinking water during gestation and lactation, from GD 0 until postnatal day 21 (PND 21). Cd/Zn doses and the manner of administration were chosen on the basis of our previous research group [22,26,53] and of available literature [3]. The dams (*n* = 6 per group) were allowed to deliver. The day of parturition was designated as post-natal day zero (PND0). The body weight progression was recorded on PND1, PND4, PND7, PND14, PND21, PND35, PND49, PND63 and PND70. Some male offspring were sacrificed at PND21. Others were weaned at PND 21 and housed in groups up to three/cage with access to the laboratory diet and drinking water, and were euthanized at adult age (PND 70).

### 4.2. Sample Collection

At PND 21, male pups were weighed then placed in a closed wooden box where high levels of ether were introduced and the anesthesia was attenuated by inhalation. Blood was taken by cardiac puncture in a heparinized tube. Plasma was separated from the blood cells by centrifugation at 5000 rpm for 10 min and stored at −20 °C. Femurs were carefully removed and weighed. Plasma and femurs were treated as described below. At PND 70, final body weight of each animal was recorded and rats were euthanized after anesthesia. The femurs were removed, cleared of adhering connective tissues, and weighed. Distal epiphysis was stored in ETOH 70% for microstructure analysis.

### 4.3. Analytical Procedures

#### 4.3.1. μCT Analysisy

Trabecular bone volume and skeletal microarchitecture of the femur were measured by high-resolution micro-CT scanner using specific software (Skyscan 1172 scanner, Aartselaar, Belgium). Briefly, each scan was performed with a source voltage of 70 kV, a current of 141 μA, a rotation step of 0.6°, and a full rotation of over 180°, with a 1 mm aluminum filter for beam hardening reduction. The pixel size was 17.67 μm, and the exposure time was 20 min. Three-dimensional (3D) microstructural image data were reconstructed using NRecon software (SkyScan). Morphometric parameters were calculated using the SkyScan CT Analyzer (CTAn) software for trabecular bone in the femur. Semi-automated contouring was used to select the region of interest (ROI) in the trabecular bone within the distal femur. The volume of interest (VOI) started at a distance of 0.77 mm from the lower end of the growth plate and extended distally for 400 cross sections (2 mm in height) comprising of trabecular bone and the marrow cavity. The bone volume fraction (%BV/TV), trabecular number (Tb. N), trabecular thickness (Tb.Th), and trabecular separation (Tb.Sp) were measured and the bone mineral density was determined according to the guidelines for assessment of bone microstructure in rodents using micro-CT [54].

#### 4.3.2. Histomorphometric Study

Bone histomorphomertry was assessed in two dimensions by means of histology. Femur samples, which did not contain fluorochrome labeling, were fixed in formalin 10%, and decalcified in nitric acid 10% for 24 h then in EDTA 10% solution at 4 °C under stirring for about three weeks. The decalcifying solution (EDTA 10%) was changed every three days [55]. Tissues were dehydrated in successive grades of ethanol series (70° and 95°) and finally cleared in xylene. Samples were then embedded in paraffin and sectioned at 3 μm longitudinally through the entire bone. The obtained sections were stained with Hariss hematoxylin and eosin Y 0.25%. Photomicrographs were taken under light microscopy by a digital microscope camera at ×20 magnification for assessment of total bone and diaphysis length and diaphysis width and at ×100 magnification for assessment of the lengths and areas of the different chondrocyte zones in the proximal and distal epiphyses. All parameters were measured using HistoLab software, and the delineation was based on cell morphology as described by previous data [27,56]. The value for each litter represents the mean of two sections per femur, and *n* = 6 were performed for each group.

#### 4.3.3. Measurement of Bone Mass Parameter (%BV/TV)

Determination of the mass parameter (%BV/TV) was performed on the histological sections of the distal femurs taken from pups at PND21, using an ocular lens for an optical microscope, equipped with a grid containing 10 vertical and horizontal lines. A region of interest (ROI) was defined on the metaphysis which contained largely trabecular bone. The method was to count the bone lamellae observed inside the field and overlapping the small lines at ×10 magnification.

Samples recording data were blind to the treatment group. Then, the percentage of bone mass (BV/TV %) was calculated as the number of trabecular bone relative to the number of optical fields defined in the ROI.

#### 4.3.4. Measurement of Cadmium, Zinc and Calcium Concentrations

Femurs destined for mineral analyses, previously weighed (W; wet weight), were oven-dried (60◦C) to obtain dry weight (DW). Then, the dried femurs were ashed, to obtain ashed weight (AW), in the muffle furnace at 650 °C overnight and for 72 h respectively for PND21 and PND70 samples. Ashed femurs were digested with concentrated nitric acid (Merck, 65%) at 120 °C. When fumes were white and the solution was completely clear, the samples were cooled to room temperature and the tubes were filled to 5 mL with ultra-pure water [53]. Individual samples were analyzed to determine Zn and Cd concentrations. In addition, calcium (Ca) was analyzed in femur samples. These measures were implemented by flame atomic absorption spectrometry using a ZEEnit 700 (Analytik, Jena, Germany) equipped with deuterium and Zeeman background correction. Samples were analyzed in triplicate, and the variation coefficient was usually less than 10%.

On the basis of the obtained weights (W, DW, and AW), the following calculations were made [57,58].

– Percentage of nonorganic components

(Wnon org. comp. = AW; % of non org. comp. = Wnon org. comp. ×100/W).

– Percentage of organic components content

(Worg. comp. = DW − AW; % of org. comp. = Worg. comp. ×100/W).

– Ash weight to dry weight ratio (AW/DW).

#### 4.3.5. Gene Expression

Total RNA from the rat femur was extracted using TRIzol (Sigma Aldrich, ST. Louis, MO, USA) according to the manufacturer’s instructions. Then, RNA was isolated with the reagent (Qiagen Oligotex mRNA Purification Kits) according to the manufacturer’s protocol. RNA quality was ensured by spectrophotometric analysis (OD260/OD280 absorption ratio >1.8) and agarose gel electrophoresis. A total amount of 1.5–2 μg of total RNA was reverse transcribed in a 20-μL reaction mixture using random hexamer primers (Roche) and 200 U of M-MuLV HRT (Fermentas, Vilnius, Lithuania), 0.5 mMdNTPs (Roche), and 19 M-MulV RT buffer as described in Banni et al. [59]. Briefly, the RNA was denatured by heating for 5 min at 70 °C, cooled on ice, and incubated with a reverse transcriptase reaction mixture. For reverse transcription, tubes were incubated at 42 °C for 60 min, followed by rapid cooling. The volume of the RT mixture was raised to 100 μL with nuclease-free distilled water, and 6 μL was used for amplification of the gene targets. The messenger RNA (mRNA) abundances of the genes encoding Runx2, colα1, ALP, Oc, ZnT5, ZIP2 and MT-1 were evaluated in multiplex TaqMan assays according to reference [59].

Probes and primer pairs (Table 4) were designed using Beacon Designer v3.0 (Premier Biosoft International, Inc.). Briefly, cDNA was amplified in the presence of 1× iQ™ Multiplex Powermix, 0.3 μM each primer, and 0.1 μM each probe (Table 4) in a final volume of 10 μL. Relative expression data were geometrically normalized to 18S ribosomal RNA (rRNA) (X01117.1), an invariant actin isotype (V01217), and glyceraldehyde-3-phosphate dehydrogenase (NM_017008.4) [60]. A specific triplex TaqMan assay was developed to amplify 0.25 ng of RNA reverse-transcribed to cDNA in the presence of 0.1 μM of each dual-labeled probe (hexachlorofluorescein/BH1 for actin, Texas Red/BH2 for 18S rRNA, and Hex/BH2 for Gapdh) and 0.1, 0.4, and 0.4 μM of forward and reverse primers, respectively, for 18S rRNA, actin, and Gapdh (Table 4). For all TaqMan assays, the thermal protocol was as follows: 30 s at 95 °C, followed by 40 cycles of 10 s at 95 °C and 20 s at 60 °C [59]. qRT-PCR was performed with four biological replicates and three technical replicates.

#### 4.3.6. Measurement of Osteocalcin and IGF-1 in the Plasma

Plasma concentrations of total osteocalcin and IGF-1 were determined by ELISA using the osteocalcin (Cat.#MK126) and IGF-1 (Cat 22-IG1MS-E01) immunoassay kit. ELISA steps were performed according to the protocol provided with the ELISA kit. The limit of detection was set to 0.029 ng/mL.

#### 4.3.7. Statistical Analysis

The results are expressed as means ± SEM, and differences among the experimental groups were assessed by one-way ANOVA, followed by a protected least significant difference Fisher’s test. Values were considered statistically significant when *p* < 0.05. The statistical analysis program used was Statview (Statview 4.57 Software). To calculate the normalized relative gene expression levels (fold induction), data were analyzed using the relative expression software tool (REST), in which the mathematical model used is based on mean threshold cycle differences between the sample and the control group [60]. For each analyzed target gene, a median PCR efficiency value obtained from at least four different experiments was used. REST was also utilized to perform a randomization test with a pairwise reallocation in order to assess the statistical significance of the differences in expression between the control and treated samples.

## Figures and Tables

**Table 1:** Cadmium (Cd) and zinc (Zn) levels in femur male pups from mother’s rats treated with Cd and/or Zn during gestation and lactation periods.

		Groups			
		C	Cd	Zn	Cd+Zn
PND21	Cd (µg/g dry weight)	nd	0.08 ± 0.03 ab	nd	0.04 ± 0.01 abc
	Zn (µg/g dry weight)	131.80 ± 6.93	70.97 ± 15.71 a	128.56 ± 8.59 b	89.79 ± 10.24 abc

Means ± SEM from six samples in each groups; a *p* < 0.01 significant difference compared to the control group (C); b *p* < 0.01: significant difference compared to the Cd group (Cd); c *p* < 0.01: significant difference compared to the Zn group (Zn).

**Table 2:** Developmental parameters of male pups from mother rats treated with Cd and/or Zn during gestation and lactation periods.

	Age	C	Cd	Zn	Cd + Zn
Relative femur weight (g/100g)	PND 21	0.799 ± 0.06	0.777 ± 0.08	0.755 ± 0.11	0.758 ± 0.10
	PND70	0.514 ± 0.06	0.473 ± 0.05	0.493 ± 0.05	0.506 ± 0.10
Cranio-caudal length	PND21	13.711 ± 0.06	12.477 ± 0.08 a*	13.288 ± 0.05 b	13.333 ± 0.06 b

Values are expressed as mean ± SEM from 12 to 18 animals from each group (2–3 male per litter was measured). a* *p* < 0.01 significant difference compared to the control group (C); b *p* < 0.05 significant difference compared to the Cd group (Cd).

**Table 3:** Organic (% CO), nonorganic (% CNO) content and ash weight to dry weight ratio (AW/DR) femur of male pups from control females and pups from females exposed to Cd and/or Zn during gestation and lactation.

		Groups			
		C	Cd	Zn	Cd+Zn
PND21	% CO	22.58 ± 2.39	22.40 ± 2.36	22.04 ± 2.38	22.28 ± 2.11
	% CNO	5.37 ± 1.08	6.64 ± 1.47	5.42 ± 1.11	6.09 ± 0.76
	AW/DR	0.19 ± 0.02	0.22 ± 0.02	0.21 ± 0.03	0.21 ± 0.008
PND70	% CO	25.22 ± 3.07	25.75 ± 4.02	28.82 ± 1.01	26.81 ± 1.49
	% CNO	23.56 ± 3.73	21.94 ± 3.57	22.59 ± 1.38	24.48 ± 2.33
	AW/DR	0.48 ± 0.05	0.46 ± 0.06	0.44 ± 0.01	0.46 ± 0.02

Means ± SEM from six samples in each groups. No significant differences have been observed between the four groups at PND 21 and PND70.

**Table 4:** Primers used for realtime PCR.

Genes	Primers Sequence for qPCR Amplification (5′-3′)
Runx2	F-TGCTTCATTCGCCTCACAA R-CTTGCTGTCCTCCTGGAGAAA
ALP	F-TCCGTGGGTCGGATTCCT R-GCCGGCCCAAGAGAGAAA
Osteocalcin	F-ATGAGGACCCTCTCTCTG R-TGCCAGGTCAGAGAGGC
Colα-1	F-GAGGATATTAATCGCATCCAGGCTTT R-CATGGATGTGGTGTTGTTGCA
ZnT5	F-GCTCTGCTCTTTGGAAACTTCTG R-CCTGGTGTGCTGCTCTGTTC
ZIP2	F-GATGCATATGACTGCTGAA R-AAGATCGGCACTGGACC
MT-1	F-ACCCCAACTGCTCCTG(C/T)(T/G)CC R-AGGTGTACGGCAAGACTCTG
β-Actin	F-CTGGGAGTGGTTTGAGGTGT R-CTATGCAGGTGGGAGGATGT
Gapdh	F-AGACAGCCGCATCTTCTTGT R-TACTCAGCACCAGCATCACC
18S	F-GTTGGTGGAGCGATTTGTCT R-GGCCTCACTAAACCATCCAA
